# Best evidence summary for the assessment and management of psychosocial distress in patients with coronary heart disease

**DOI:** 10.3389/fcvm.2026.1738470

**Published:** 2026-03-02

**Authors:** Yaxian Xu, Ying Xing, Wei Xu, Zhenbo He, Maodan Zhang, Lixia Chen

**Affiliations:** Department of Nursing, The Fourth Affiliated Hospital of School of Medicine, and International School of Medicine, International Institutes of Medicine, Zhejiang University, Yiwu, China

**Keywords:** best evidence, coronary heart disease, management, prevention, psychological stress

## Abstract

**Objective:**

Psychological distress significantly affects the progression of coronary heart disease (CHD), functional recovery and overall well-being. This study aims to establish an evidence-based foundation for clinical practice by systematically retrieving and synthesizing the best evidence on the assessment and management of psychosocial distress in CHD.

**Methods:**

According to the ‘5S’ evidence model, a top-down search strategy was conducted to collect relevant evidence, including guidelines, best practice, evidence summaries, expert consensus, systematic reviews or Meta-analyses. The search for this study covered the period from the database inception through September 10, 2025. Two reviewers independently screened and assessed the literature, then synthesized the evidence using the JBI evidence grading and recommendation system.

**Results:**

A total of 21 articles were ultimately included, comprising 3 guidelines, 5 expert consensuses, 1 clinical decision, and 12 systematic reviews. This study summarizes 24 pieces of evidence across five aspects of social and psychological distress in patients with CHD: Personnel Qualifications and Team Composition, Psychological Assessment, Psychological Interventions, Continuity of Care and Follow-up Management, and Identification and Referral of Severe Issues.

**Conclusion:**

This study systematically synthesizes the best available evidence from five core domains concerning early assessment and intervention for psychosocial distress in patients with CHD. The findings may inform the development of individualized psychological support strategies in clinical settings, facilitating timely alleviation of negative emotions and improving patient engagement in cardiac rehabilitation.

**Systematic Review Registration:**

http://ebn.nursing.fudan.edu.cn/resource/summary, identifier ES20244245.

## 1 Introduction

Coronary heart disease (CHD) is a disease characterized by psychosomatic interactions, often accompanied by psychosocial distress such as anxiety, depression, post-traumatic stress disorder, fear, and perceived stress (1, 2). Studies have shown that psychological distress is not only closely related to disease progression and decreased quality of life, but also increases the risk of recurrence of cardiac events (3, 4). Anxiety and depression are widely recognized as independent risk factors for CHD, while CHD itself exacerbates these symptoms, creating a vicious cycle (5, 6). Therefore, psychosocial distress is one of the key factors affecting cardiac rehabilitation in patients with CHD.

There is currently a lack of standardized protocols for psychological assessment, intervention, timing, and monitoring in patients with CHD. This poses significant challenges for the early identification and management of psychosocial distress. Pharmacological treatment can partially alleviate anxiety and depression symptoms in CHD patients. However, given concerns about drug side effects, addiction risks, and potential drug interactions, non-pharmacological interventions hold significant value in early identification and safety considerations (7). Strong evidence demonstrates that integrating psychological care into CHD management improves emotional regulation, reduces symptom burden, and enhances quality of life (8, 9). In this context, nurses, who have the most direct patient contact, play a central role in advancing psychological care and are critical to enhancing patient outcomes (10). However, in current clinical practice, early identification of psychological problems in patients with CHD remains insufficient (11). Therefore, this study adopts the “best evidence summary” method, aiming to extract, synthesize and transform the current high-level evidence on the evaluation and intervention of psychological distress in patients with CHD, and form an evidence-based recommendation that can directly guide clinical nursing practice.

## 2 Method

### 2.1 Search strategy

According to the “5S” evidence resource model (12), evidence retrieval is searched from the top-down. The databases searched included: BMJ Best Practice, Up To Date, National Institute of Health and Clinical Excellence (NICE), National Guideline Clearinghouse (NGC), Guideline International Network (GIN), Scottish Intercollegiate Guidelines Network (SIGN), European Society of Cardiology (ESC), American Heart Association (AHA), Cochrane Library,Web of Science, PubMed, Australian JBI Proof-Based Health Care Database, China Biology Medicine, China Knowledge Resource Integrated Database (CNKI), Wanfang and VIP. Corresponding search terms included combinations of: “Coronary Disease/coronary heart disease/Percutaneous Coronary Intervention/percutaneous coronary interventions” “stress/psychological/psychological stress/psychological status/negative emotions/fear/anxiety/depression” “guideline/systematic review/meta-analysis/evidence summary/consensus/clinical decision/best practice”. The search encompassed records from database inception to 10 September 2025.

### 2.2 Literature inclusion and exclusion criteria

Inclusion criteria were as follows: (a) the study population consisted of patients with CHD, (b) the study content involved nursing care related to adverse psychological stress responses in these patients, (c) outcome measures included psychological stress, (d) evidence types included expert consensus, systematic reviews, guidelines, evidence summaries, and meta-analyses, (e) language was either Chinese or English.

Exclusion criteria were as follows: (a) literature with incomplete content or for which the full text could not be obtained, (b) literature type was conference abstract, guideline interpretation, research plan/proposal or the old guide that has been replaced.

### 2.3 Literature screening and quality evaluation

The design and implementation of this study followed the PRISMA guidelines. Literature screening was conducted independently by two researchers. Duplicates were merged and removed using EndNote X9, and title/abstract screening as well as full-text screening were assisted by the software. Any inconsistencies were resolved by discussion or arbitration by a third researcher.

The included literature underwent a structured quality assessment. Clinical guidelines were appraised using the Appraisal of Guidelines for Research and Evaluation II (AGREE II) instrument (13). Systematic reviews, expert consensuses, and evidence summaries were evaluated using the corresponding critical appraisal tools from the Australian JBI Centre for Evidence-Based Health Care (2016) (14). Following independent assessments, the two researchers compared their results, and any discrepancies were resolved through discussion or by consulting a third researcher to reach consensus.

### 2.4 Evidence grading and recommendation formulation

The included evidence was appraised using the JBI Evidence Grading and Recommendation System, with evidence levels graded from 1 to 5 (15). Under the guidance of the FAME structure, a comprehensive evaluation was conducted, and the recommendation strength was categorized as either Grade A (strong recommendation) or Grade B (weak recommendation).

## 3 Results

### 3.1 General characteristics of the included literature

This study initially retrieved 1,845 publications. After reviewing titles, abstracts, and full texts, duplicate and non-conforming literature were excluded. A total of 21 articles were ultimately included, comprising 3 guidelines (16–18), 5 expert consensuses (19–23), 1 clinical decision (24), and 12 systematic reviews (9, 25–35). The study selection process is presented in Supplementary Figure 1, and the basic characteristics of the included literature are presented in Table 1.

**Table 1:** General information of the included literature.

Included literature	Year	Literature sources	Type of evidence	Topic of the literature
(16)	2025	Wanfang	Guideline	Depression and anxiety in coronary artery disease
(17)	2024	ESC	Guideline	Chronic coronary syndromes management
(18)	2019	GIN	Guideline	depression screening and treatment after acute coronary syndrome
(19)	2025	ESC	Expert consensus	Mental health and cardiovascular disease
(20)	2021	CNKI	Expert consensus	Hospitalized patients with CHD
(21)	2020	CNKI	Expert consensus	Psychological prescription for patients with cardiovascular disease
(22)	2019	CNKI	Expert consensus	Rehabilitation treatment of integrative medicine for stable CHD
(23)	2023	Wanfang	Expert consensus	Diagnosis and treatment of stable coronary artery disease combined with mental disorders in primary care
(24)	2021	UpToDate	Evidence summary	Psychosocial factors in coronary and cerebral vascular disease
(25)	2024	Cochrane	Systematic review	Psychological interventions for depression and anxiety in patients with CHD, heart failure or atrial fibrillation.
(26)	2017	Cochrane	Systematic review	Psychological interventions for CHD
(27)	2025	Web of science	Systematic review	Effectiveness of psychological interventions in reducing post-traumatic stress among post-myocardial infarction patients
(28)	2025	Web of science	Systematic review	The Influence of eHealth Stress Management Interventions in Patients with Cardiovascular Disease
(9)	2022	Web of science	Systematic review	Efficacy of psychological interventions on clinical outcomes of coronary artery disease
(29)	2021	Web of science	Systematic review	Effectiveness of cognitive behavioral therapy-based interventions on health outcomes in patients with CHD
(30)	2024	PubMed	Systematic review	Effects of mHealth interventions on quality of life, anxiety, and depression in patients with CHD
(31)	2024	PubMed	Systematic review	Mindfulness therapy for patients with CHD
(32)	2023	PubMed	Systematic review	Efficacy of cognitive behavior therapy in reducing depression among patients with CHD
(33)	2022	PubMed	Systematic review	Anxiety following myocardial infarction
(34)	2021	PubMed	Systematic review	Mindfulness-based interventions for patients with CHD
(35)	2020	CNKI	Systematic review	Efficacy of Chinese medicine care in patients

### 3.2 Quality evaluation results of the included literature

#### 3.2.1 Quality evaluation results of the guidelines

This study included a total of 3 clinical practice guidelines. The standardized domain scores and overall quality assessment results of these guidelines are presented in Table 2.

**Table 2:** Methodological quality evaluation results of the guidelines.

Included literature	Percentage of field standardization %						≥60% field number (*n*)	≥30% field number (*n*)	Recommendation level
	①	②	③	④	⑤	⑥			
(16)	83.3%	72.2%	69.8%	77.8%	60.4%	83.3%	6	6	A
(17)	97.2%	80.6%	82.3%	91.7%	72.9%	83.3%	6	6	A
(18)	86.1%	86.1%	82.3%	87.5%	75.0%	79.2%	6	6	A

(1) Scopes and objects (2) Participant (3) Rigour of the guidelines (4) Clarity of guidelines (5) Application of guidelines (6) Independence of the guide.

#### 3.2.2 Quality evaluation results of expert consensuses

5 expert consensus articles were independently evaluated by two evaluators according to JBI expert opinion quality evaluation tool, and the overall quality of the articles was medium or high, so they were approved for inclusion. Detailed quality evaluation contents are shown in Table 3.

**Table 3:** Methodological quality evaluation of expert consensus.

Expert consensus	Evaluation entry						Overall quality
	①	②	③	④	⑤	⑥	
(19)	Yes	Yes	Yes	Yes	Yes	Yes	High
(20)	Yes	Yes	Yes	Yes	Yes	Yes	High
(21)	Yes	Yes	Yes	Yes	Yes	Yes	High
(22)	Yes	Yes	Yes	Yes	Yes	Yes	High
(23)	Yes	Yes	Yes	Yes	Yes	Yes	High

(1) Is the source of the opinion clearly stated? (2) Are the opinions from influential experts in the field? (3) Are the opinions presented centered on the interests of the people involved in the study? (5) Is the stated conclusion based on the results of the analysis? Are opinions expressed logically? (5) Whether to refer to other existing literature? (6) Are there any inconsistencies between the opinions presented and the previous literature?

#### 3.2.3 Quality evaluation results of the evidence summary

For the 1 included evidence summary, we employed the JBI Critical Appraisal Tool for Evidence Summaries for methodological quality assessment. The overall quality was evaluated as high.

#### 3.2.4 Quality evaluation results of systematic reviews and meta-analyses

This study included 12 systematic reviews and meta-analyses, among which 5 studies rated all items as “yes”. 3 studies (27, 28, 33) did not discuss publication bias. 2 studies (29, 34) lacked comprehensive search strategies, and 1 study (32) did not have two or more reviewers independently assess study quality. 1 Chinese study (31) lacked a comprehensive search strategy and did not specify whether the criteria for evaluating studies were appropriate.

### 3.3 Evidence summary results

Through the synthesis of evidence related to psychological stress assessment and intervention in patients with CHD, 24 evidence-based recommendations were ultimately formulated across five domains: personnel qualifications and team composition, psychological assessment, psychological intervention, continuity of care and follow-up management, and identification and referral of severe issues. Details are presented in Table 4.

**Table 4:** Summary of best evidence for the psychosocial distress in patients with CHD.

Evidence items		Evidence content	Level of evidence	Recommended level
Personnel qualifications and team composition		1. A multidisciplinary team comprising cardiologists, psychiatrists, nurses, cardiac rehabilitation therapists, and psychotherapists is recommended to implement a collaborative mental health model (19–21, 27).	5b	A
		2. Personnel performing psychological assessment and intervention within the team must possess relevant qualification certifications or have completed systematic psychological education and training with assessment (18–20, 22, 27).	5b	A
		3. It is recommended that cardiovascular nurses who have undergone systematic training and passed assessment lead the implementation of basic psychological assessment and intervention (27).	1a	B
Psychological assessment	Assessment principles	4. Psychological assessment should adhere to the principle of continuous and dynamic evaluation, specifically following the cycle of “assessment, intervention, and re-assessment” (20, 24).	5b	B
	Assessment timing	5. It is recommended to implement psychological screening for patients during the early stages of CHD management (16, 20, 24).	5b	A
		6. Healthcare professionals should integrate continuous monitoring of psychological stress throughout the entire course of CHD, with the perioperative period requiring heightened attention (24).	5b	B
		7. For patients with access to follow-up care, assessment and intervention should be extended and sustained into the outpatient rehabilitation phase (16, 21, 24).	5b	B
	Assessment tools	8. The use of validated measurement tools is recommended for screening psychological distress, depression, and anxiety in patients with CHD (16, 17, 19, 25).	1a	A
		9. A stepped screening approach is recommended: brief tools should be used for initial rapid identification, followed by further assessment of screen-positive individuals using standardized scales to determine severity (19, 20, 23).	1a	A
Psychological interventions	Intervention models	10. It is recommended to establish and strengthen a tripartite collaborative mechanism involving hospitals, families, and communities throughout the entire psychological intervention process (23).	1b	A
		11. It is recommended to adopt an individualized, stepped-care approach, developing intervention strategies through shared decision-making that incorporates psychological methods, patient preferences, and symptom severity (16–20, 24, 26).	5b	A
	Intervention formats	12. Psychological interventions should be tailored to the patient's specific circumstances and needs, utilizing various formats such as individual counseling, group sessions, remote support, and mobile health technologies (16, 21, 25, 27, 28, 31–33).	1b	A
	Intervention content	13. Nursing staff should routinely incorporate fundamental psychological support techniques, such as active listening, empathy, acceptance, and affirmation, into patient care (20, 26).	1a	A
		14. The use of cognitive behavioral therapy (CBT) is recommended, implementing structured CBT courses that include techniques such as cognitive restructuring, problem-solving, relaxation training, and emotion regulation (9, 16–18, 21, 24, 26, 29, 32).	1b	A
		15. Mindfulness-based relaxation training is recommended, incorporating structured practices such as body scan, seated meditation, and mindful movement (21, 27, 31, 33, 34).	1b	A
		16. It is recommended to teach patients stress management techniques, including muscle relaxation, environmental adjustment, attitude training, controlled breathing, and word repetition exercises (24, 33).	1b	B
		17. It is recommended to instruct patients in pressure management skills, including pressure identification, cognitive restructuring, emotion management, and relaxation training (21, 33).	1b	B
		18. It is recommended to integrate traditional Chinese medicine (TCM) nursing with TCM emotion theory, applying methods such as desire fulfillment therapy, mental introspection therapy, cognitive guidance, emotional restriction therapy, and five-element music therapy (21, 23, 35).	5b	B
		19. Develop internet-based, individualized psychological interventions and provide systematic support for them using decision-aid tools, personalized information, and diversified resources (16, 28).	1a	A
		20. It is recommended to fortify the patient support system by bolstering family support, fostering peer mutual assistance, and broadening social engagement (20).	5b	B
Continuity of care and follow-up management		21. It is recommended that available psychological support resources are communicated to patients and families before discharge, and that follow-up records are documented to monitor psychological status over time (16, 17, 20, 21, 23, 24, 33).	1a	A
		22. It is recommended that nurses collaborate with patients and their families at discharge to develop a follow-up plan, establishing diversified follow-up channels such as in-person clinic visits, telephone calls, video consultations, and mobile applications (20), Association., 2020, (21, 23, 24).	5b	A
Identification and referral of severe issues		23. For patients with complex or severe psychological issues, psychiatric physicians and specialist nurses should be promptly invited to participate in consultations (20–22), Psycho-cardiology Group, 2023, (24).	5b	B
		24. Patients identified through assessment as having moderate to severe psychological disorders should be provided with prompt referrals and assisted with transfer to a psychiatric department for specialized intervention (16, 20–24, 32).	5b	A

## 4 Discussion

### 4.1 Multidisciplinary team collaboration and nursing capacity building serve as the core support of the psychological care system

Multidisciplinary team is the key way to improve the psychological nursing system for patients with CHD. The ideal team should include professionals in the fields of cardiology, nursing, cardiac rehabilitation and psychiatry (24). As first-line managers, nurses play an important role in early identification of patients with post-traumatic stress disorder, anxiety, depression and other negative emotions. However, at present, nurses generally lack systematic psychological assessment and intervention training, and their core competence is limited (36–38). At the same time, it should be noted that non-professionals must obtain corresponding qualifications for psychological intervention, which constitutes an institutional barrier to ability transformation (39). Therefore, it is necessary to establish a hierarchical psychological nursing training system for cardiovascular nurses. Through the combination of short-term special training and long-term systematic education, it is necessary to focus on improving their core competencies such as psychological assessment, basic intervention and therapeutic communication, and to support nurses to obtain psychological related qualifications through institutionalized incentives to ensure the effectiveness of nursing practice norms.

### 4.2 Assessment of negative psychological states in patients with CHD should adhere to the principles of continuity and dynamic evaluation

CHD as a major negative health event, tends to exert a persistent psychological impact on patients and trigger intense stress responses (40). Research indicates that patients with coronary heart disease exhibit a higher incidence of psychosocial distress during the perioperative period, and associated negative emotions may persist into the post-discharge phase (41, 50). However, current research primarily focuses on the diagnosis and treatment of acute-phase CHD, with insufficient attention paid to patients’ mental health during the post-discharge rehabilitation phase (11, 42). This results in inadequate intervention for adverse health outcomes caused by psychological distress. Therefore, this study recommends integrating psychological assessment into the routine management process for CHD. Specifically, initial screening should be initiated at an early stage to promptly identify adverse psychological stress. Building on this, a dynamic assessment process spanning the entire disease course should be established, enabling targeted interventions based on the psychological characteristics of patients at each stage, ultimately aiming to alleviate psychological distress and enhance participation and adherence to cardiac rehabilitation.

In the process of implementing dynamic assessment, in addition to paying attention to the immediate psychological state of patients, stable individual factors should also be integrated. Studies have shown that personality traits (Neuroticism and Conscientiousness) are systematically associated with patients ‘ self-health evaluation, psychological distress and disease management response (43, 44). For example, individuals with high Neuroticism may require earlier emotional regulation support, while those with low Conscientiousness may rely on more structured behavioral supervision and follow-up. Recent studies have further revealed that among patients with CHD, individuals with lower levels of Conscientiousness may be more vulnerable when facing long-term rehabilitation management and lifestyle adjustments, with corresponding increases in psychological risk (42). At present, the relevant evidence on CHD and personality traits mainly focuses on cross-sectional studies, but these findings provide an important theoretical basis for the construction of a precise nursing model of personality adaptability ‘ in the future (43). Future research can further verify whether the inclusion of personality traits in dynamic assessment can effectively identify high-risk groups, and explore the feasibility and effectiveness of providing enhanced emotional regulation training or more structured behavioral supervision.

### 4.3 Appropriate application of psychological care techniques to alleviate adverse psychological stress in patients with CHD

The rational application of psychological care techniques serves as an effective means to alleviate psychosocial distress in patients with CHD (45). Intervention formats should be flexibly selected and combined based on the patient's disease stage, psychological needs, and resource availability, such as individual counseling, group support, and remote guidance. This approach enhances the accessibility and adaptability of psychological support (25).

In terms of intervention content, psychological nursing should form a hierarchical content system of “basic support-structured intervention-continuous reinforcement”. Basic support technologies such as listening, empathy and affirmation are the cornerstones for establishing treatment alliances and promoting communication and trust (33, 46). On this basis, structured intervention methods with empirical support can be introduced for specific problems of patients. Among them, cognitive behavioral therapy is recommended as a first-line psychological intervention program for CHD patients with depression or anxiety through cognitive reconstruction and behavioral activation, which can help alleviate emotional symptoms and improve self-management ability (47). Mindfulness training, stress management, and Internet-based individualized interventions have also shown positive effects in improving emotional regulation and treatment compliance (28, 31, 33, 48). Looking forward to the future, while systematically implementing evidence-based programs, we can actively explore the innovative integration of localized physical and mental adjustment techniques such as traditional Chinese medicine emotional conditioning and modern psychological nursing models, and it is expected to develop an integrated intervention path that is more in line with China ’s cultural context and patients ‘ health beliefs.

### 4.4 Improving follow-up systems and referral mechanisms

Establishing a systematic continuity of care framework is essential for ensuring the continuity and comprehensiveness of psychological support for patients (49). During discharge preparation, accessible psychological support resources should be systematically organized for patients and families (20). Standardized follow-up records should be established to support ongoing dynamic monitoring and intervention. In the post-discharge follow-up phase, diversified follow-up support channels need to be developed (17, 33). Nurses should collaborate with patients and their families at discharge to develop individualized follow-up plans. These plans may use outpatient visits, telephone calls, video consultations, and mobile apps to continuously monitor psychological status and changes, improving the timeliness and precision of interventions.

When screening reveals complex or severe psychological problems, promptly activate a cross-disciplinary collaboration (24, 32). Include psychiatrists and specialist nurses in joint consultations to formulate intervention plans. Establish clear referral pathways for patients with moderate to severe disorders to psychiatry for systematic evaluation and treatment. This supports the complete management cycle of screening, identification, referral, and intervention.

## 5 Limitation

This study synthesizes evidence on psychological care for CHD patients, yet several limitations exist. First, the geographical representativeness of the included literature may be limited, as a considerable proportion of studies originated from China. This may affect the generalizability of the findings to other cultural and healthcare settings. Second, the restriction to Chinese and English literature could introduce language bias and omit relevant evidence. Third, there are few clinical practice guidelines for psychological intervention in patients with CHD, which may limit the comprehensiveness and depth of evidence summary. Future research needs to improve the comprehensiveness and situational adaptability of evidence by expanding the search scope, dynamically updating and evidence-based application.

## 6 Conclusion

This study synthesizes 24 pieces of evidence across five domains of social and psychological distress in CHD patients, personnel qualifications and team composition, psychological assessment, psychological interventions, continuity of care and follow-up management, identification and referral of severe issues. This study provides a structured basis for systematically strengthening psychological nursing in clinical nursing of coronary heart disease. Based on the current evidence, it is recommended that medical staff should integrate patient preferences, disease staging, and local resource accessibility when formulating psychological care plans, so as to selectively apply relevant evidence. At present, high-quality randomized controlled trials for psychological distress in patients with coronary heart disease are still insufficient, which restricts the accuracy of intervention strategies. Therefore, future research should focus on developing and verifying more targeted psychological intervention strategies to optimize the mental health outcomes of this group.

## Data Availability

The original contributions presented in the study are included in the article/Supplementary Material, further inquiries can be directed to the corresponding author.
