# Supplementary material for: Best evidence summary for the assessment and management of psychosocial distress in patients with coronary heart disease
Source: Front Cardiovasc Med. 2026 Mar 2;13:1738470. doi: 10.3389/fcvm.2026.1738470 (PMC12989332; doi:10.3389/fcvm.2026.1738470)
Supplement: Supplementary file 1 [file Image1.pdf]

Supplementary Material

1 Supplementary Figures

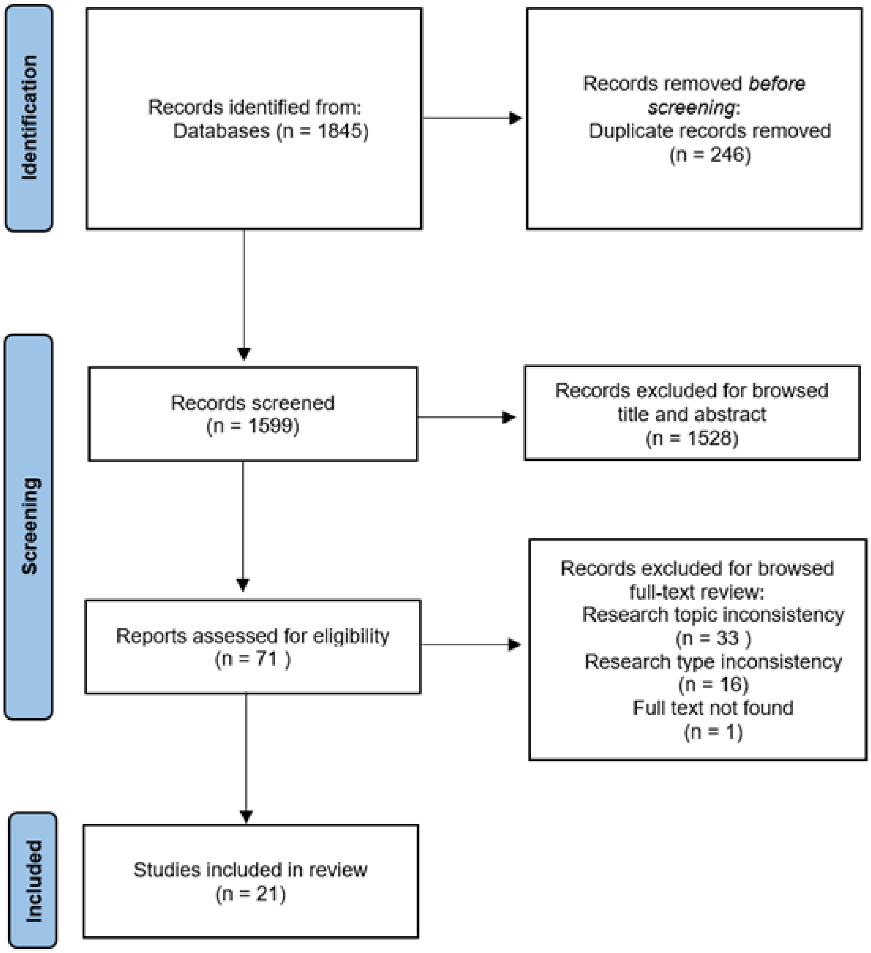

Supplementary Figure 1. Flow diagram of literature search.
